# Asymmetric dimethylarginine (ADMA) is identified as a potential biomarker of insulin resistance in skeletal muscle

**DOI:** 10.1038/s41598-018-20549-0

**Published:** 2018-02-01

**Authors:** Woojung Lee, Hyo Jung Lee, Han Byul Jang, Hyo-Jin Kim, Hyo-Jeong Ban, Kwang Youl Kim, Moon Suk Nam, Joo Sun Choi, Kyung-Tae Lee, Seong Beom Cho, Sang Ick Park, Hye-Ja Lee

**Affiliations:** 1 Center for Biomedical Sciences, National Institute of Health, Osong Health Technology Administration Complex, Chungcheongbuk-do, South Korea; 2 Division of Food Microbiology, Ministry of Food and Drug Safety, Osong Health Technology Administration Complex, Chungcheongbuk-do, South Korea; 3 Center for Genome Science, National Institute of Health, Osong Health Technology Administration Complex, Chungcheongbuk-do, South Korea; 4 Department of Clinical Pharmacology, Inha University Hospital, Incheon, South Korea; 5 Department of Internal Medicine, Inha University College of Medicine, Incheon, South Korea; 6 Department of Home Economics Education, College of Education, Kyungnam University, Changwon-si, Gyeongsangnam-do, South Korea; 7 Department of Phamaceutical Biochemistry, College of pharmacy, Kyung Hee University, Seoul, South Korea

## Abstract

To unravel metabolic determinats of insulin resistance, we performed a targeted metabolomics analysis in Korean Children-Adolescent Cohort Study (KoCAS, n = 430). Sixty-seven metabolites were associated with insulin resistance in adolescents and the association also found in an adult population (KoGES, n = 2,485). Functional interactions of metabolites with gene/proteins using biological pathway with insulin resistance were not identified biological significance and regulatory effects of asymmetric dimethylarginine (ADMA). However, ADMA showed a higher association with adolescent obesity (P < 0.001) and adult diabetes (P = 0.007) and decreased after obesity intervention program. Functional studies in cellular and mouse models demonstrated that an accumulation of ADMA is associated with the regulation of obesity-induced insulin resistance in skeletal muscle. ADMA treatment inhibited dimethylarginine-dimethylaminohydrolase (DDAH) activity and mRNA expression in insulin resistance muscle cell. Moreover, the treatment led to decrease of phosphorylation of insulin receptor (IR), AKT, and GLUT4 but increase of protein-tyrosine phosphatase 1B (PTP1B). Accordingly, increased ADMA significantly inhibited glucose uptake in myotube cell. We suggest that accumulation of ADMA is associated with modulation of insulin signaling and insulin resistance. ADMA might expand the possibilities of new therapeutic target for functional and clinical implications in the control of energy and metabolic homeostasis in humans.

## Introduction

Nitric oxide (NO) is synthesized from the guanidine group of arginine via the enzyme family NO synthases (NOS) which consist of three isoforms^1,2^. One of these, the constitutive endothelial NOS (eNOS) enzyme, is stimulated by insulin and cofactors such as tetrahydrobiopterin^3,4^. Conversely, NOS is inhibited by the endogenous asymmetric dimethylarginine (ADMA) and L-monomethylarginine (LMMA)^3,5,6^. Dimethylarginines are increasingly recognized as important markers or factors of endothelial dysfunction and cardiovascular disease^5,7^. In particular, ADMA concentration increases with diabetes, hypertension, hypercholesterolemia, and aging^8–11^.

ADMA is produced by the proteolysis of intracellular proteins that are post-translationally modified by type І protein *N*-arginine methyltransferase (PRMT)^5,8^. It is metabolized by NG, NG-dimethylarginine dimethylaminohydrolase (DDAH) to L-citrulline and dimethylamine, after uptake from the circulation^5,8^. *DDAH1* and *DDAH2*, the two isoforms of DDAH, regulate the levels of ADMA in blood and tissue^6,8,12^. ADMA is both exported from its site of origin, and imported from the plasma at distant sites, by cationic amino acid transporters (CATs) in exchange for arginine and other cationic amino acids (CAAs)^11,13^.

Insulin resistance is one of the major characteristics of type 2 diabetes mellitus (T2D) and also occurs with obesity, hypertension and cardiovascular disease^14,15^. A major characteristic of T2D is insulin resistance in skeletal muscle^16,17^. Skeletal muscle accounts for ~40% of body mass and contributes ~80% of whole body insulin stimulated glucose disposal^14^.

We could not fully explain the precise mechanism of insulin resistance related to obesity. However, altered adipokines (such as adiponectin, leptin and resistin), increased circulating pro-inflammatiory cytokines (such as interleukin-1ß, interleukin-6, tumor necrosis factor α), reactive oxygen species and glycated proteins-mediated glucotoxicity and elevated free fatty acids-induced lipotoxicity are presumed to cause the impairment of insulin signaling and hence insulin resistance^14,16^. Insulin binds to the insulin receptor tyrosine kinase and activates the receptor and then phosphorylates the Insulin receptor substrate (IRS). Phosphrylated IRS activates the phosphatidylinositol 3-kinase and Akt pathway and is connected to translocation GLUT4 to cell surface, ultimately cellular glucose uptake^18,19^. The tyrosine phosphorylated insulin receptor β and IRS-1 is targeted and hydrolyzed by protein tyrosine phosphatase 1B (PTP1B), which acts to regulate insulin signaling, negatively^20–22^.

Here, we conducted a study in obese adolescent subjects who were undergoing insulin resistance to determine whether plasma metabolites such as BCAAs, AAA, acycarnitines^23–25^, are higher in obese adolescents than in normal-weight adolescents and there are novel different metabolites in each other. Also, comparisons in plasma targeted metabolites concentrations were made between prediabetes and diabetes subjects and a group of normal-adult subjects. In addition, we performed a functional study to decide whether the difference of plasma metabolites account for insulin resistance in trait-determining cell types and mouse models.

## Results

### Relations of plasma ADMA concentrations and insulin resistance

**Figure 1:**
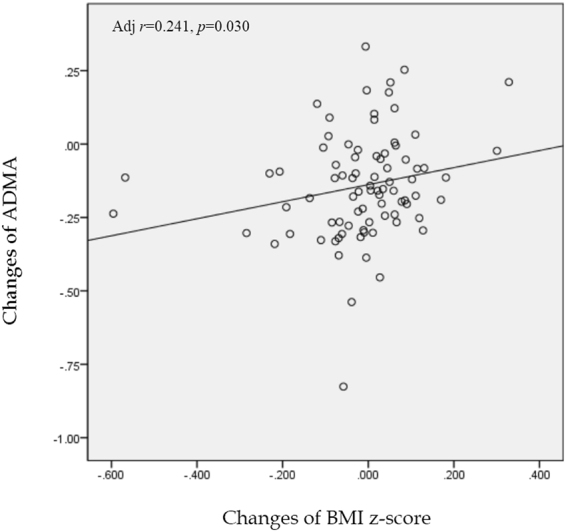
Comparison of changes of ADMA and BMI z-score in morbid adolescent after 10-week obesity intervention program. The analyses were performed using Pearson’s partial correlation (using covariates) analyses. Covariates included age and sex.

To unravel metabolic determinants of insulin resistance predicting obesity and diabetes, we performed a targeted metabolomic analysis in a Korean adolescents (Table 1) and quantified 141 metabolites. Of these, 67 metabolites such as acylcarnitines, amino acids, biogenic amines, glycerophospholipids, sphingolipids, and hexose, were associated with insulin resistance in adolescents (*p* < 3.55E-04; Bonferroni correction, Supplementary Table S1) and 52 metabolites of them were associated with HOMA-IR in an adult population (*p* < 0.05, Table 1 and Supplementary Table S2). To find common biological associations between insulin resistance and these metabolites, we performed both pathway analysis using the Pathway Studio and pathway enrichment analysis using ConsensusPathDB and KEGG pathway resources (Supplementary Fig. S1). Sub-cellular network analysis demonstrated the metabolites shared biological significance underlying canonical signaling pathways such as insulin, AMPK, PI3-Akt, and type 2 diabetes signaling pathway, but not ADMA. We evaluated the association between ADMA levels and risk of obesity and diabetes using multivariate-adjusted ORs to predict admissible functional effects of ADMA (Table 2). After adjustment for age and sex, an increased ADMA was associated with the risk of severe obesity compared to normal weight adolescents (OR = 1.67, *p* = 0.0002). Also, prediabetes and diabetes adults had higher levels of plasma ADMA than normal subjects (OR = 1.44, *p* = 0.021; OR = 1.45, *p* = 0.006; respectively). Furthermore, plasma ADMA concentrations were fell with 10-week intervention program (*p* < 0.001, Supplementary Table S3) in morbid adolescents and a positive correlation between change of ADMA and BMI z-score was observed during this period (*r* = 0.24, *p* = 0.030, Fig. 1).

**Table 1:** Clinical characteristics of study subjects.

	KoCAS (n = 430)	KoGES (n = 2485)
Age (years)	13.9 ± 0.60	56.8 ± 8.98
Boys or men (%)	54.4	48.2
Height (cm)	163.7 ± 7.44	159.8 ± 9.14
Weight (kg)	72.0 ± 21.1	62.7 ± 10.4
BMI (kg/m^2^)	26.7 ± 6.78	24.5 ± 3.24
Fat mass (kg)	25.4 ± 15.3	16.6 ± 5.61
Waist circumference (cm)	84.7 ± 17.8	85.2 ± 9.07
Hip circumference (cm)	100.6 ± 12.0	91.8 ± 5.44
WHR (waist-to-hip ratio)	0.83 ± 0.09	0.93 ± 0.08
Systolic blood pressure (mmHg)	121.0 ± 51.7	117.9 ± 16.8
Diastolic blood pressure (mmHg)	75.6 ± 9.18	78.7 ± 10.3
Glucose (mg/dL)	94.6 ± 11.0	95.7 ± 19.8
AST (IU/L)	23.4 ± 13.2	26.4 ± 21.0
ALT (IU/L)	24.6 ± 28.0	24.3 ± 20.4
Total cholesterol (mg/dL)	166.1 ± 28.2	193.8 ± 36.3
Triglyceride (mg/dL)	99.4 ± 64.1	146.6 ± 116.4
HDL-cholesterol (mg/dL)	49.9 ± 9.80	43.9 ± 10.3
Insulin (µIU/mL)	19.9 ± 18.3	7.73 ± 3.76
HOMA-IR	4.77 ± 4.89	1.86 ± 1.09
ADMA	0.55 ± 0.15	0.60 ± 0.18

Values are expressed as the mean ± SD or %.

BMI, body mass index; HDL, high density lipoprotein; HOMA-IR, homeostasis model assessment insulin resistance; ADMA, asymmetric dimethylarginine.

**Table 2:** Odds ratio (95%CI) for risk of Obesity and type 2 diabetes by plasma ADMA level.

Study sample		Cases, N	ADMA		
			OR	(95% CI)	p-value
KoCAS	Weight status				
	Normal weight	171	1		
	Overweight or Obesity	98	1.67	(0.64–4.33)	0.293
	Severe Obesity	161	6.48	(2.44–17.24)	**<0.001**
KoGES	Diabetes status				
	Normal	989	1		
	Prediabetes	975	1.27	(0.99–1.62)	0.058
	Diabetes	517	1.56	(1.13–2.16)	**0.007**

ADMA, Asymmetric dimethylarginine.

ADMA is log transformed before analysis.

Adjusted for age and sex in KoCAS.

Adjusted for age, sex, and region in KoGES.

### Glucose tolerance, serum concentration of glucose and insulin, and insulin resistance in ob/ob mice

**Figure 2:**
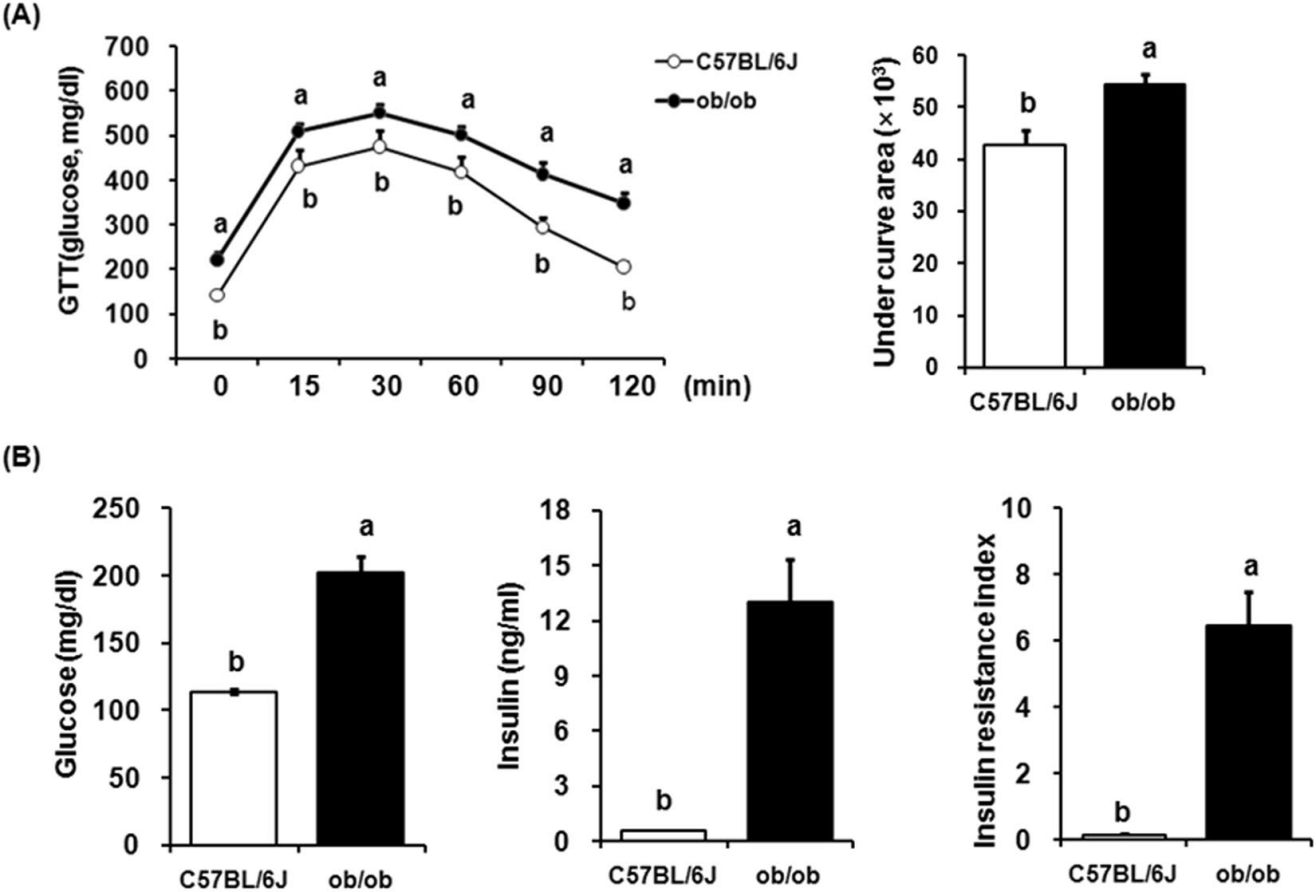
Metabolic disorders in ob/ob mice. (**A**) Glucose tolerance test (GTT) at 3 weeks after high fat diet. Mice were challenged with an oral feeding of glucose (2 g/kg body weight). Blood samples were obtained from the tail at 0, 15, 30, 60, 90, and 120 minutes after glucose administration to measure glucose concentration. (**B**) Plasma glucose, insulin, and insulin resistance were measured from 10 week-old male *ob/ob* and age-matched lean mice. Insulin resistance index calculated with HOMA index. ^*^Significantly different between groups at p < 0.05.

Three weeks after high fat diet, glucose tolerance was significantly impaired in ob/ob mice compared with normal C56BL/6 J mice (Fig. 2). The area under the curve for blood glucose was significantly higher for the ob/ob mice than for the C57BL/6 J mice (Fig. 2A). The ob/ob mice fed high fat diet definitely showed hyperglycemia, hyperinsulinemia, and insulin resistance. Five weeks after the high fat diet, plasma glucose, insulin and insulin resistance (HOMA index) also increased significantly in the ob/ob mice compared with C56BL/6 J mice (Fig. 2B).

### Increased ADMA levels in ob/ob mice

**Figure 3:**
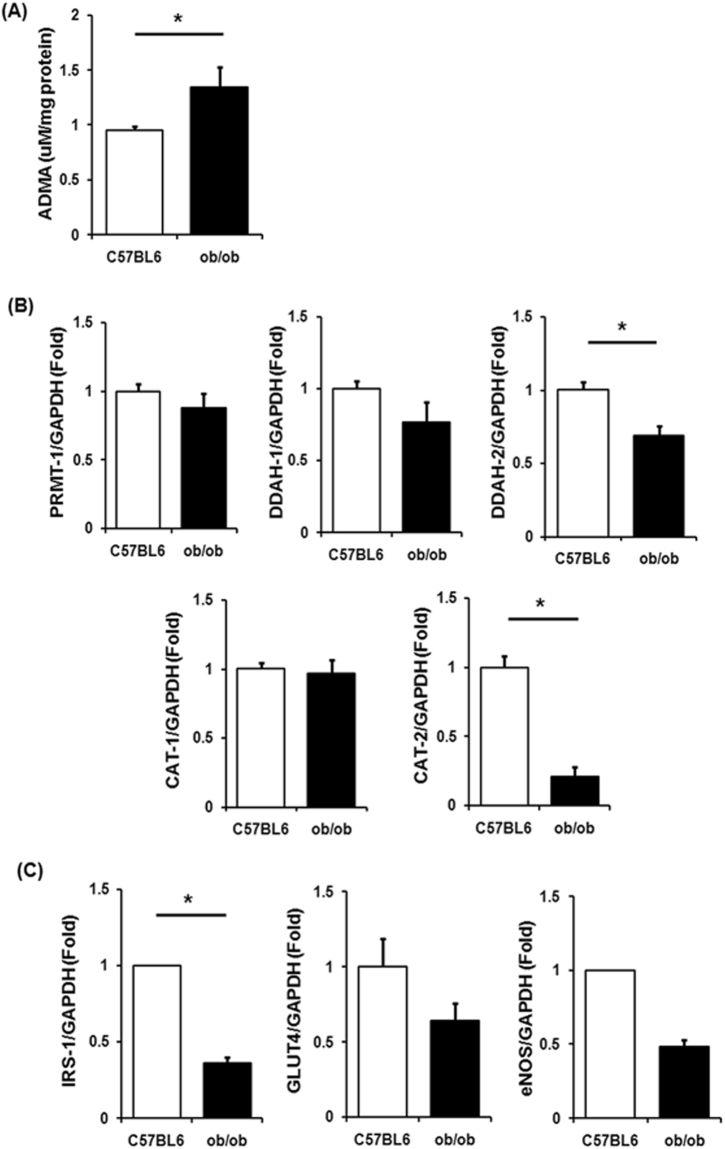
Change of ADMA levels and ADMA metabolism in *ob/ob* mice. (**A**) ADMA concentration in muscle. (**B**,**C**) Quantitative real time PCR was performed from muscle tissues of 10 week-old male *ob/ob* and age-matched lean mice. ^*^Significantly different between groups at p < 0.05.

Based on the results from population data, we tried to identify the mechanism underlying the relationship between insulin resistance and ADMA concentrations by using mouse and cell model which simulate muscle, the major organ of glucose metabolism. ADMA concentrations and its metabolism involved gene expressions in mouse model are shown in Fig. 3. In skeletal muscle of ob/ob mice, ADMA levels were about 30% higher than that of control C57BL6 mice despite not significantly difference in gene expression levels of PRMT-1 (Fig. 3A and B). On the contrary, gene expression levels of DDAHs and CATs, the enzymes which metabolize and transport ADMA respectively, decreased with obesity (Fig. 3B). Specifically, ob/ob mice showed 79% decreased CAT-2 expression levels compared to normal C57BL6 mice.

The ob/ob mice showed significantly lower mRNA expression of genes involved in insulin signaling and glucose uptake, IRS-1 and GLUT4, compared to normal mice (65% and 36% decreases, respectively; Fig. 3C). The gene expression level of endothelial nitric oxide synthase (eNOS), which is inhibited by ADMA, also showed 52% decrease in ob/ob mouse model.

### Accumulation of ADMA in Palmitate-induced insulin resistant C2C12 myotubes

**Figure 4:**
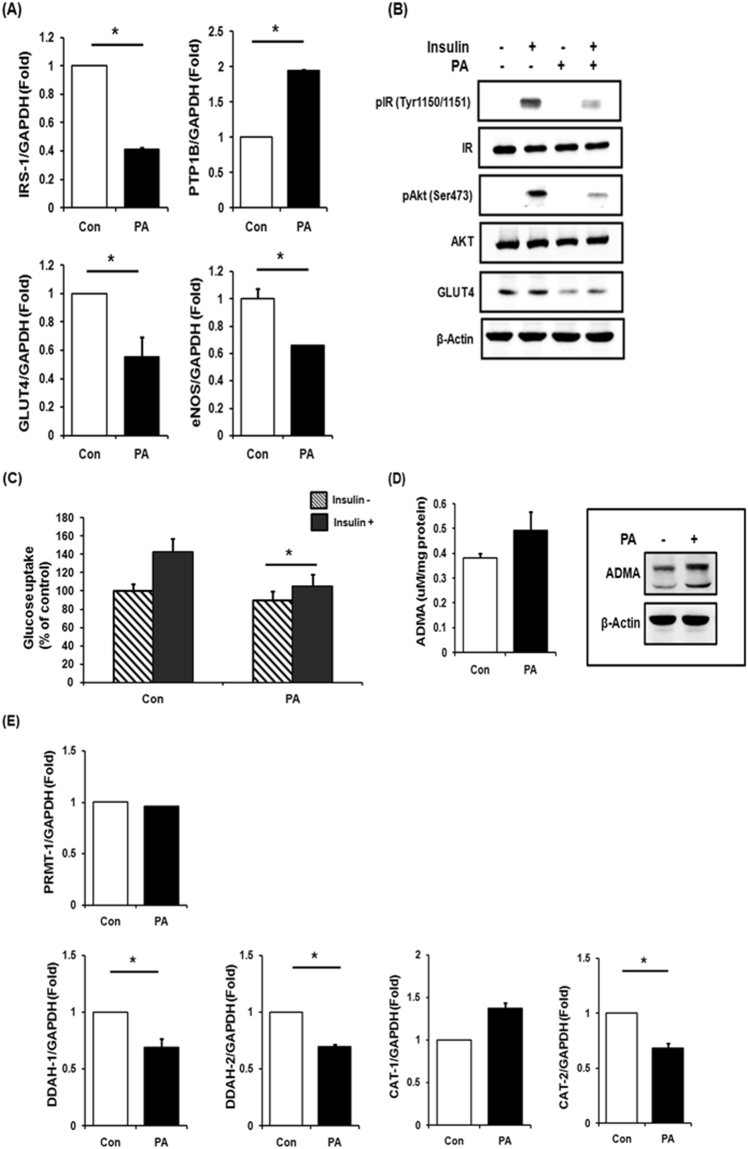
Palmitate induced insulin resistance and increased ADMA levels. (**A**) Quantitative real time PCR was performed in C2C12 mouse myotubes treated with palmitate (0.75 mM) for 18hr. (**B**) Insulin was treated for 20 min and followed by western blot. (**C**) Basal and insulin-stimulated glucose uptake assessed. (**D**) Whole lysate was extracted from palmitate-treated C2C12 mouse myotubes and LC-MS/MS or western blot were performed. (**E**) The C2C12 mouse myotubes treated with palmitate (18hr, 0.75 mM), followed by quantitative real time PCR. ^*^Significantly different between groups at p < 0.05.

Further, C2C12 myotubes were treated with palmitate (0.75 mM, 18 h) to induce insulin resistance. Compared to normal cells, palmitate-treated cells showed decrease in mRNA expression levels of IRS-1, GLUT-4, eNOS, but increase in PTP1B (Fig. 4A), which means impaired insulin signaling. With insulin treatment, palmitate-induced insulin resistant cells showed significantly lower protein expressions of GLUT4 and activated form of insulin receptor and Akt (Fig. 4B). Along with reduced expression of GLUT4, insulin and palmitate-treated myotubes showed significantly lower glucose uptake than control cells (26% reduction; Fig. 4C). In these palmitate-induced insulin resistant cells, ADMA concentrations were higher than their counterpart cells (Fig. 4D). Consistently, gene expression levels of DDAHs and CAT-2 in insulin-resistant cells were significantly lower than control cells, which might lead to accumulation of ADMA in insulin-resistant cells, but not PRMT-1 (Fig. 4E).

### ADMA inhibits DDAH expression and activity in C2C12 myotubes

**Figure 5:**
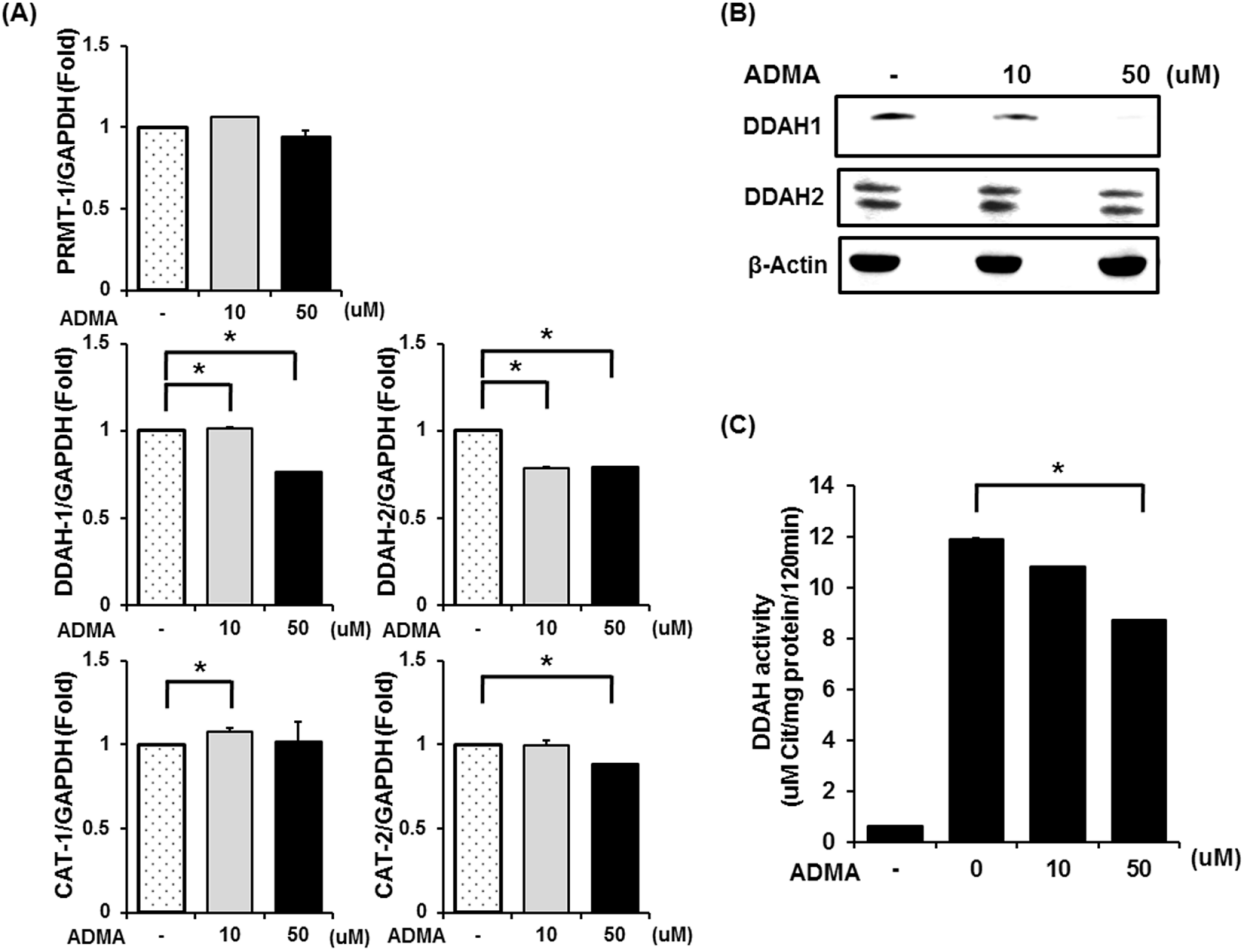
Excessive ADMA decreased DDAH. (**A**,**B**) 10uM or 50uM ADMA was treated in C2C12 mouse myotubes for 18hr was performed in whole cell lysates by quantitative real time PCR or western blot. (**C**) DDAH activity assessed in C2C12 mouse myotubes treated with ADMA 10uM or 50uM for18hr. ^*^Significantly different between groups at p < 0.05.

Then, we treated ADMA on C2C12 myotubes and measured ADMA metabolism-involved genes expression levels and enzyme activity in order to investigate mechanisms underlying increased ADMA concentrations in ob/ob mouse muscle (Fig. 5). Compared to normal myotubes, ADMA treated C2C12 myotubes showed significant decrease in mRNA expressions of ADMA metabolizing enzyme DDAHs at 50 μM of ADMA concentrations. As for ADMA transporting enzyme CAT-2, mRNA expressions of it was also decreased at 50 μM. However, there was no significant change in gene expression of ADMA producing enzyme PRMT-1 (Fig. 5A).

Protein expression of DDAH-1 showed consistent responses to ADMA concentrations; as ADMA concentrations increased, expression of DDAH-1 and DDAH-2 enzymes gradually decreased (Fig. 5B). Along with reduced expressions of DDAHs depending on ADMA concentrations, we found decrease in capacity of DDAHs, which was measured by conversion from ADMA to L-citrulline (Fig. 5C). Specifically, ADMA concentration at 50 μM led to significantly decreased conversion capacity of DDAHs than normal cells (26% of reduced converting capacity).

### ADMA induced insulin resistance and glucose uptake dysfuction in C2C12 myotubes

**Figure 6:**
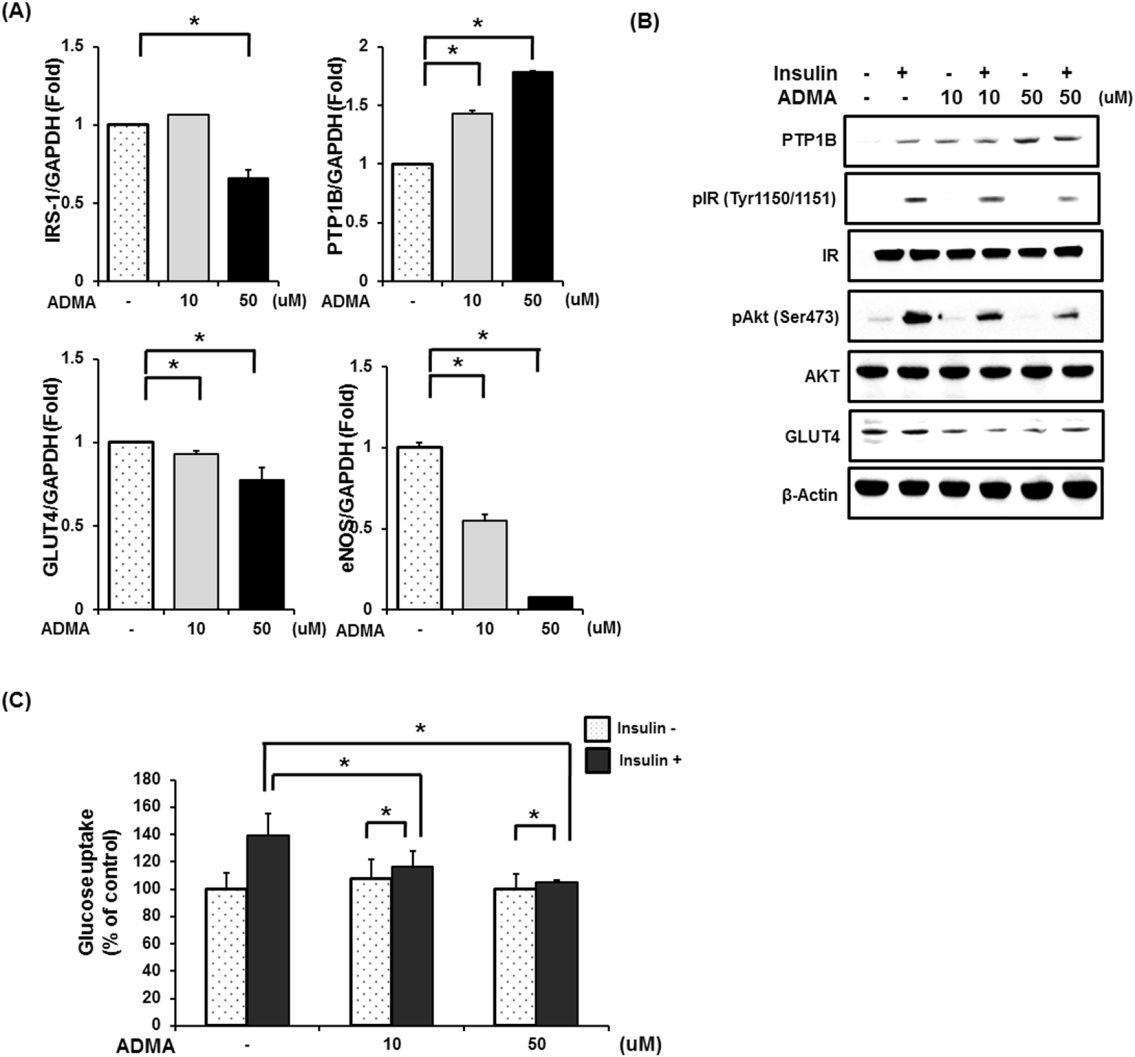
Excessive ADMA induced insulin resistance. (**A**,**B**) 10uM or 50uM ADMA was treated in C2C12 mouse myotubes for 18hr and insulin signaling-related factors was measured in whole cell lysates by quantitative real time PCR or western blot. (**C**) Basal and insulin-stimulated glucose uptake assessed. ^*^Significantly different between groups at p < 0.05.

Next, we measured gene expression levels of IRS-1, PTP1B, and GLUT4 with a goal for investigating the effect of accumulated ADMA resulted by inhibition of ADMA metabolism and transport on insulin signaling and glucose uptake (Fig. 6). Compared to normal cells, ADMA treated C2C12 myotubes showed significant reduced gene expression levels of IRS-1 and GLUT4, which suggests impaired insulin signaling and glucose uptake (Fig. 6A). To be specific, at 50 μM of ADMA concentration, gene expression levels of IRS-1 and GLUT-4 were 66% and 78% of those without ADMA treatment, respectively. The enzyme which is inhibited by ADMA, eNOS, also showed significantly reduced gene expression levels at 10 and 50 μM of ADMA concentration (54% and 1% of normal expression levels, respectively). On the other hand, gene expressions of PTP1B, which inhibits insulin signaling via de-phospholylation of insulin receptor, increased with ADMA concentrations (40% and 80% increases compared to no ADMA treatment).

Similar to gene expressions, protein expression levels of PTP1B and GLUT4 changed depending on treated ADMA concentrations (Fig. 6B); protein levels of PTP1B in ADMA-treated cells increased with ADMA concentrations compared to normal cells contrary to reduction in GLUT4 protein expression at high ADMA concentration. When it comes to insulin receptor and Akt, which are phosphorylated with insulin presence, phosphorylation levels of them also decreased with ADMA concentrations.

Consistently, ADMA-treated C2C12 myotubes showed disturbed glucose uptake at insulin presence (Fig. 6C). When insulin and ADMA treated at the same time, glucose uptake by C2C12 myotubes significantly decreased compared to glucose uptake with insulin treatment alone (16% and 24% reduction, at 10 and 50 μM of ADMA treatment, respectively).

## Discussion

Based on the Korean Children-Adolescent Cohort Study (KoCAS) and adult Ansan and Ansung cohort study (KoGES), we observed significantly positive correlation between plasma ADMA concentration and HOMA-IR, the surrogate marker of insulin resistance widely used in epidemiologic studies^15^. Moreover, we observed the decrease of plasma level of ADMA after intervention program in morbid obese adolescents and positive correlation between change in ADMA and BMI z-score. In experiments using muscle tissues and C2C12 myotubes, we found the association between impaired insulin signaling and glucose uptake and accumulation of ADMA, which seems to be induced by disturbance in expression and activity of enzymes which are involved in ADMA metabolism. These results implicate the possibility of ADMA as a potential biomarker of insulin resistance in skeletal muscle, the major organ of insulin-stimulated glucose disposal^14^.

Since ADMA inhibits synthesis of nitric oxide (NO) which is essential for maintenance of endothelial function, raised ADMA concentration has been well known as a marker of cardiovascular diseases (CVD)^5,8^. NO, the main vaso-relaxing factor produced by endothelial cells through the action of endothelial NO synthase (eNOS), is a potent inhibitor of platelet function, all of which endow NO with the anti-atherosclerotic properties^8^. In clinical studies, those with cardiovascular diseases including coronary artery disease, chronic heart failure, pulmonary hypertension, and stroke showed raised plasma ADMA concentrations compared to healthy individuals^5^. The possible molecular mechanism that elevated ADMA causes to CVD is the eNOS uncoupling, leading to reduced NO bioavailability and increased hydrogen peroxide production in pathological conditions such as atherosclerosis and type 2 diabetes mellitus (T2D)^2^. Therefore, elevated ADMA concentration might be a biomarker of CVD risk as well as insulin resistance or T2D^7,11^. McLaughlin and colleagues^26^ reported that plasma ADMA levels were higher in obese and/or insulin resistant middle-aged women compared to their counterparts and elevated ADMA levels fell with weight loss. Consistently, in the present study, we observed significantly risky relations between ADMA concentrations and insulin resistance in obese adolescents and diabetic adults, even in otherwise healthy subjects, which implies that ADMA could be an early marker of insulin resistance. In addition, higher ADMA levels but lower eNOS gene expression levels were observed in ob/ob mice skeletal muscles compared to those of control group.

ADMA is synthesized when arginine residues in proteins are methylated by the action of protein arginine methyltransferases (PRMTs). On the other hand, dimethylarginine dimethylaminohydrolase (DDAH) degrades ADMA to dimethylamine and L-citrulline^2^. Thus, changes in DDAH activity may contribute to elevated ADMA levels in various diseases^2^. According to a study by Sydow and colleagues^27^, transgenic mice of which overexpressed human DDAH-I had lower plasma ADMA at all ages by comparison to wild-type littermates. Similarly, in this study, ob/ob mice showed lower gene expression levels of DDAHs and CATs (transporting enzyme) compared to their counterparts, whereas those of PRMTs were not different between two groups. These results indicate that accumulation of ADMA in skeletal muscle is due to decrease in degradation of ADMA by DDAH. In addition, accumulated ADMA seems to interfere with DDAH activity in that ADMA-treated C2C12 myotubes showed decrease in DDAHs capacity of conversion from ADMA to L-Citrulline depending on treated ADMA concentration.

Since skeletal muscle is the major site of glucose uptake in the postprandial state in humans, a major characteristic of T2D is insulin resistance in skeletal muscle^16,17^. When the insulin receptor tyrosine kinase is activated with insulin binding, the downstream docking protein IRS is phophorylated, which activates the phosphatidylinositol 3-kinase and Akt pathway and is connected to translocate GLUT4 vesicles and subsequently entry of glucose into the cell^18,19^. On the other hand, the protein tyrosine phosphatase 1B (PTP1B) hydrolyzes the phophorylated insulin receptor β and IRS-1^20–22^, therefore it functions to regulate insulin signaling, negatively^20–22^. Collectively, insulin signaling and its subsequent glucose uptake could be measured through these key molecules. In the present study, ADMA treatment on C2C12 myotubes led to reduced expression levels of IRS-1, activated form of insulin receptor and Akt, and GLUT4. In contrast, expression level of the protein tyrosine phosphatase 1B (PTP1B) increased with ADMA treatment. These changes in gene expression levels resulted in decreased actual glucose uptake by C2C12 myotubes with insulin treatment compared to control cells. Taken together, elevated ADMA concentration might induce attenuate insulin signaling and subsequent glucose uptake in the skeletal muscle. These results are similar to C2C12 myotubes with palmitate treatment, the other route to induce insulin resistance^22^, which indicate ADMA has a role of inducing insulin resistance. Additionally, the fact that palmitate-treated C2C12 myotubes showed increased ADMA concentration might propose the vicious cycle of insulin resistance and ADMA accumulation.

In conclusion, we identified ADMA as a potential biomarker of insulin resistance in skeletal muscle which could lead to T2D. Although some studies have proposed the possibility of ADMA as a biomarker of insulin resistance or T2D, they mainly focused on the relationship between ADMA and endothelial dysfunction^7,11^ or reported simple association between elevated ADMA concentration and insulin resistance with small sample size^26^. In contrast, we analyzed human plasma samples as well as determined the relationship between accumulated ADMA and insulin resistance with using cell and mouse models; ADMA treatment on C2C12 myotubes inhibits its degrading enzyme DDAH thus leads to accumulation of it, and in the end could attenuate insulin signaling and subsequent glucose uptake in skeletal muscle, the major organ of glucose disposal^14^. Additionally, we measured ADMA levels in cells and tissues by using LC-MS/MS methods, the more sensitive method to the traditional analyzing method NMR^28^. Lastly, we observed that the association between increase in ADMA concentration and insulin resistance regardless of its induction route: both skeletal muscle tissues from ob/ob mice fed high-fat diet and palmitate-treated C2C12 myotubes showed elevated ADMA concentrations compared to their counterparts.

Nevertheless, there are some limitations to this study. First, the association between elevated ADMA concentrations and insulin resistance might be due to obesity, the common cause of them. As the relationship between obesity and insulin resistance is well known, our subjects with the highest BMI percentile had the highest average HOMA-IR as well as plasma concentrations of insulin in adolescents of this study including intervention program. This is due to the fact that elevated free fatty acid, and inflammatory cytokine concentrations, all of which are characteristics of obese people can induce insulin resistance^16^. However, we did functional studies with using cell and mouse models in order to identify the effect of elevated ADMA concentration on insulin resistance and underneath mechanism. Secondly, our subjects were otherwise healthy and young adolescents, and this study is cross-sectional. As for age and sex, the other factor which could affect HOMA-IR, there was no difference among the groups, and we adjusted for age and sex when calculating odds ratio. Lastly, longitudinal cohort studies are needed to demonstrate whether elevated ADMA concentrations at baseline can predict future risk of T2D (regardless of their baseline BMI) and the basic reason underlying elevated ADMA concentrations in obese people.

## Method

### Population study

#### Subjects

For this study, data were obtained from the Korean Children and Adolescents Study (KoCAS) and Korean Genome Epidemiology Study (KoGES) conducted by the Korea National institute of Health (KNIH). The 430 adolescents, aged 13–15 years, were recruited from Seoul and Kyunggi province in 2012 as part of the KoCAS. The KoGES subjects consisted of 2,485 individuals, aged 40–69 years and with available information of diabetes diagnosis and metabolite data, from Ansan and Ansung city between 2005 and 2006^29^. In the short-term obesity intervention study, a total of 91 morbid obese adolescents aged between 12 and 16 years old with BMI ≥ 99^th^ percentile or BMI ≥ 30 kg/m^2^ were included. The basic intervention program including minimal exercise and nutrition education was performed three times during 10 weeks. The study protocol was approved by the institutional review board of Seoul-Paik Hospital, Inje University, and the Korea Center for Disease Control and Prevention (IIT-2012-092 and 2017-02-06-P-A). Informed consent was obtained from the adolescent’s parents. The methods were carried out in accordance with the approved guidelines.

#### Biochemical measurement

Triglyceride (TG), total cholesterol, high-density lipoprotein-cholesterol (HDL-C), and glucose levels were measured using enzymatic assays and an autoanalyzer (model 7600II; Hitachi, Tokyo, Japan). Fasting serum insulin was measured using a Roche E170 analyzer (Roche Diagnostics, Mannheim, Germany). The insulin resistance index was calculated as HOMA-IR, the clinical usefulness of homeostasis model assessment of insulin resistance^30^.

#### Targeted metabolite profiling

Plasma samples were analyzed with the AbsoluteIDQ™ p180 Kit using the protocol described in the AbsoluteIDQ user manual (Biocrates Life Sciences AG, Innsbruck, Austria). Amino acids and biogenic amines including plasma asymmetric dimethyl-arginine (ADMA) were quantified by stable isotope dilution in LC-MS/MS. Acylcarnitines, lipids, and hexoses were quantified by flow injection analysis (FIA-MS/MS) using a ABI 4000 Q-Trap mass spectrometer (Applied Biosystems/MDS Sciex, Foster city, CA) at Inha University Hospital Clinical Trial Center. In total, 141 metabolites passed the above criteria, and the final metabolomics dataset contained a Targeted metabolites data for adult were provided by the Korea Biobank (MetIDQ p180).

#### Statistical analysis

Statistical analyses were performed with SPSS software (version 21.0; SPSS Inc., Chicago, IL, USA). Variables were examined for normality, and those that were not normally distributed were normalized by logarithmic transformation for analyses. In performing association tests for insulin resistance, HOMA-IR, linear regression analysis was conducted after adjustment for age and sex in adolescents and age, sex. In adults, the association between 67 metabolites found in adolescents and HOMA-IR was identified using linear regression models, adjusted for age, sex, and region. Logistic regression analyses were performed to calculate odds ratios of the risk of childhood obesity and adult diabetes after adjustment. The association between change of ADMA and BMI z-score was assessed using Pearson’s partial correlation analyses. All data are expressed as the mean ± standard deviation (SD). We considered a p-value of less than 0.05 to be statistically significant.

### Molecular pathway analysis

Pathway Studio 10.0 software (Ariadne Genomics, Rockville, MD) was utilized to analyze the functional interactions. It was used to identify specific gene/proteins interact with metabolites that are differentially expressed in normal and insulin resistance tissue. It provides the interpretation of biological implication from gene/protein expression data, the establishment of molecular pathways, and identification of protein interaction maps and their associations to cellular process^31^. We performed pathway enrichment analyses with insulin resistance specific gene/proteins using ConsensusPathDB^32^, ConsensusPathDB integrates interaction networks in human including complex protein-protein interaction, genetic, metabolic, biochemical pathways. We used KEGG pathway resources for biological process. Visualization of pathway mapping was performed in Cytoscape^33^. Metabolites are mapped to light-blue square nodes, and biological pathway or disease names are mapped to grey circular nodes.

### Functional studies

#### Animals and biochemical analysis

All animal protocols were approved by the animal care and use committee of the National Institute of Health (KCDC-015-11-2A). The methods were carried out in accordance with the approved guidelines. Six-weeks of genetically male obese *ob/ob* mice and age-matched lean normal C57BL/6 J mice were purchased from the Japan SLC (Shizuoka, Japan) and housed in polycarbonate cages in a temperature-regulated (22 °C) and humidity-controlled (55%) room with a 12-h light/12-h dark cycle. Water and normal standard pellet diet were available *ad libitum* throughout the experimental period. After 1 week of adaptation, food intake was recorded daily and body weights were monitored every 3 day during the feeding period. At the end of week, mice were fasted overnight and anesthetized using CO_2_. Glucose tolerance test (GTT) was performed by intraperitoneal (i.p.) injection of glucose (1 g/kg body weight) after overnight fasting after 3 weeks of high fat diet. Glucose in a tail vein blood sample was measured at 15- or 30-min intervals for 2 h, using a glucose meter (Arkray Global Business, Inc., Kyoto, Japan). After overnight fasting, the muscle was removed from each mouse and used for LC-MS/MS, quantitative real time PCR and Western blot analysis.

#### Cell culture and treatments

C2C12 cells were obtained from ATCC and cultured in Dulbecco’s modified Eagle’s medium (DMEM) containing 10% fetal bovine serum (FBS) and 1% antibiotics. For differentiation, myoblaste were seeded in 100mm^2^ dish and cultured in same medium until 90% of confluence. After this period, the cells were differentiated in DMEM containing 2% horse serum until formation of myotubes (5 days). Myotubes were then incubated for 18 hours in DMEM containing 2% BSA and 10% FBS in absence or presence of 0.75 mM palmitate or ADMA. Subsequently, the palmitate or ADMA treated cells were stimulated with 200 nM insulin for 10 min.

#### DDAH activity measurement and 2-NBDG glucose uptake assay

DDAH activity was measured by the conversion of ADMA to L-citrulline (Tain and Baylis, 2007). In brief, protein concentration was determined by the BCA Protein Assay (Pierce Chemical, Rockford, IL) and adjusted to 20 mg/ml. The protein homogenate was preincubated with urease to eliminate interference of the analysis by urea. Standards were prepared by use of 3 to 50 mM L-citrulline. The absorption was read spectrophotometrically at 450 nm. DDAH activity is presented as millimolar L-citrulline per gram of protein per minute at 37 °C.

Myotubes were incubated for 18 h in DMEM containing 2% BSA and 10% FBS in absence or presence of 0.75 mM palmitate or ADMA. Subsequently, the palmitate or ADMA treated cells were stimulated with 200 nM insulin for 1 h. After insulin stimulation, myotubes were incubated with 50 μM 2-NBDG (Invitrogen, Carlsbad, CA) for 15 min and then washed with PBS three times to remove free 2-NBDG. The fluorescence intensity of cells containing 2-NBDG was measured on VICTOR X3 microplate reader (PerkinElmer, Waltham, MA, USA) with excitation at 485 nm and emission at 535 nm.

### Measurement of ADMA with LC-MS/MS

#### Sample Preparation

Sample cleanup for LC-MS/MS analysis was performed as follows. The calibration standard, tissue homogenate, cell lysates and cell media samples (100 μL) were transferred into a 1.5-mL eppendorf tube. Then, 10 μL of ISTD working solution and 900 μL of PBS buffer were added. The ADMA was extracted with MCX SPE cartridge (Waters) as described previously^34^. After SPE, the only 100ul eluent of final volume of 1 mL was transferred into a new glass tube and evaporated at 40 °C under N2 gas stream. The ADMA derivatization reaction was performed using PITC reagent^35^. The residue was reconstituted in 100 μL of coupling solution (ACN:pyridine:triethylamine:water, 10:5:2:3), vortexed for 30 s and added 5 μL of PITC solution. The reaction was allowed to proceed for 5 minutes at room temperature and the samples were dried under N2 at 40 °C. Before analysis the samples were reconstituted in 200 μL of mobile phase. Then, 1 μl sample was injected onto LC-MS/MS system.

#### LC-MS/MS analysis

The chromatographic separation was performed with C18 column (Hypersil gold C18, 3.0um, 2.0 × 100 mm) using a gradient with acetonitrile-water (0.1% formic acid) and with a run time of 3.5 min. The analytes were detected by electrospray ionization mass spectrometry in positive ion mode on an API 4000 triple quadrupole mass spectrometer (MDS SCIEX, Toronto, Canada). Quantification was carried out using multiple reaction monitoring (MRM) mode of the transitions *m*/*z* 338.2 → 203.2 and 344.2 → 209.2 for ADMA-PITC and ISTD, respectively. The source parameters of the mass spectrometer were optimized and maintained as follows: collision activated dissociation (CAD) gas, 4; curtain gas (CUR), 20; gas 1 (nebulizer gas), 60; gas 2 (heater gas), 60; turbo ion spray (IS) voltage, 5300 V; and source temperature, 650 °C. Other optimized compound parameters for monitoring analyte were set as follows: declustering potential (DP), 60 V; entrance potential (EP), 10 V; collision energy (CE), 22 V; and collision cell exit potential (CXP), 15 V. Calibration curves were constructed by the peak area ratios of the analyte to its corresponding ISTD by weighted (1/x) least-squares linear regression. Data processing were performed on Analyst 1.5.1 software (SCIEX).

## Electronic supplementary material

Supplementary Information
