# Supplementary material for: Asymmetric dimethylarginine (ADMA) is identified as a potential biomarker of insulin resistance in skeletal muscle
Source: Sci Rep. 2018 Feb 1;8:2133. doi: 10.1038/s41598-018-20549-0 (PMC5794993; doi:10.1038/s41598-018-20549-0)

Asymmetric dimethylarginine (ADMA) is identified as a potential biomarker of insulin resistance in skeletal muscle

Woojung Lee[1,2*], Hyo Jung Lee[1*], Han Byul Jang[1*], Hyo Jin Kim[1], Hyo-Jeong Ban[3], Kwang Youl Kim[4], Moon Suck Nam[5], Joo Sun Choi[6], Kyung-Tae Lee[7], Seong Beom Cho[3], Sang Ick Park[1], Hye-Ja Lee[1]

[1]Center for Biomedical Sciences, National Institute of Health, Osong Health Technology Administration Complex, Chungcheongbuk-do, South Korea

[2]Division of Food Microbiology, Ministry of Food and Drug Safety, Osong Health Technology Administration Complex, Chungcheongbuk-do, South Korea

[3]Center for Genome Science, National Institute of Health, Osong Health Technology Administration Complex, Chungcheongbuk-do, South Korea

[4]Department of Clinical Pharmacology, Inha University Hospital, Incheon, South Korea

[5]Department of Internal Medicine, Inha University College of Medicine, Incheon, South Korea

[6]Department of Home Economics Education, College of Education, Kyungnam University, Changwon-si, Gyeongsangnam-do, South Korea

[7]Department of Phamaceutical Biochemistry, College of pharmacy, Kyung Hee University, Seoul, South Korea

Supplementary Table S1. Linear regression between HOMA-IR and plasma metabolites in KoCAS

Metabolites	R^2	Adjusted R^2	Beta	p-value
**Acylcarnitines**				
Carnitine (C0)	0.063	0.056	0.834	6.91E-07
Decenoylcarnitine (C10:1)	0.061	0.055	-0.486	9.41E-07
Tetradecenoylcarnitine (C14:1)	0.046	0.040	-0.422	3.12E-05
Hydroxytetradecenoylcarnitine (C14:1-OH)	0.091	0.084	-0.665	8.43E-10
Tetradecadienylcarnitine (C14:2)	0.073	0.066	-0.395	5.87E-08
Hydroxytetradecadienylcarnitine (C14:2-OH)	0.043	0.036	-0.425	6.57E-05
Octadecenoylcarnitine (C18:1)	0.042	0.036	-0.567	8.01E-05
Propionylcarnitine (C3)	0.090	0.083	0.678	1.13E-09
Pimelylcarnitine (C7-DC)	0.130	0.124	-0.682	6.21E-14
Nonaylcarnitine (C9)	0.059	0.053	-0.671	1.53E-06
**Amino-acids**				
Alanine	0.119	0.113	1.220	8.95E-13
Arginine	0.088	0.082	0.538	1.47E-09
Asparagine	0.065	0.058	-0.868	3.91E-07
Aspartate	0.036	0.029	0.171	3.51E-04
Glutamate	0.236	0.230	0.802	4.29E-26
Glycine	0.090	0.084	-1.035	1.23E-09
Isoleucine	0.145	0.139	1.223	1.32E-15
Leucine	0.129	0.123	1.288	7.48E-14
Lysine	0.073	0.067	0.984	5.66E-08
Phenylalanine	0.099	0.093	1.399	1.08E-10
Proline	0.074	0.068	0.854	4.14E-08
Tryptophan	0.040	0.033	0.745	1.29E-04
Tyrosine	0.174	0.168	1.538	8.53E-19
Valine	0.186	0.180	1.750	3.33E-20
**Biogenic Amines**				
Asymmetric dimethylarginine (ADMA)	0.037	0.030	0.470	3.46E-04
Creatinine	0.052	0.045	-0.850	7.97E-06
Kynurenine	0.075	0.068	0.801	3.75E-08
alpha-Aminoadipic acid	0.083	0.077	0.444	5.19E-09
**Glycerophospholipids - Phosphatidylcholine (PC)**				
PC aa C32:1	0.058	0.051	0.409	2.08E-06
PC aa C36:1	0.042	0.035	0.545	9.37E-05
PC aa C36:3	0.072	0.065	0.912	7.39E-08
PC aa C38:0	0.047	0.040	-0.562	2.96E-05
PC aa C38:3	0.125	0.119	0.939	2.17E-13

PC aa C40:1	0.086	0.079	-1.050	2.95E-09
PC aa C40:2	0.071	0.064	-0.833	1.04E-07
PC aa C40:4	0.046	0.040	0.479	3.17E-05
PC aa C40:5	0.054	0.048	0.485	4.63E-06
PC aa C42:0	0.079	0.073	-0.702	1.25E-08
PC aa C42:1	0.081	0.074	-0.728	9.53E-09
PC aa C42:4	0.038	0.032	-0.626	1.99E-04
**Glycerophospholipids - Phosphatidylcholine (PC)**				
PC ae C30:0	0.060	0.053	-0.715	1.38E-06
PC ae C32:1	0.068	0.062	-0.849	1.77E-07
PC ae C34:0	0.045	0.039	-0.543	3.84E-05
PC ae C34:1	0.066	0.060	-0.924	2.84E-07
PC ae C34:3	0.126	0.120	-0.887	1.43E-13
PC ae C36:0	0.049	0.043	-0.547	1.47E-05
PC ae C36:2	0.095	0.089	-0.892	2.73E-10
PC ae C38:2	0.054	0.047	-0.611	5.49E-06
PC ae C40:1	0.100	0.093	-0.877	1.00E-10
PC ae C40:4	0.059	0.053	-0.854	1.48E-06
PC ae C40:5	0.055	0.049	-0.754	3.62E-06
PC ae C40:6	0.091	0.085	-0.918	7.19E-10
PC ae C42:1	0.053	0.046	-0.798	6.61E-06
PC ae C42:2	0.040	0.033	-0.632	1.52E-04
PC ae C42:3	0.104	0.098	-1.043	3.51E-11
PC ae C42:4	0.050	0.044	-0.594	1.17E-05
PC ae C42:5	0.066	0.060	-0.840	2.89E-07
PC ae C44:5	0.054	0.048	-0.596	4.91E-06
PC ae C44:6	0.053	0.047	-0.596	6.03E-06
**Glycerophospholipids - lysoPC**				
lysoPC a C17:0	0.091	0.084	-0.665	8.49E-10
lysoPC a C18:1	0.081	0.074	-0.708	9.24E-09
lysoPC a C18:2	0.056	0.049	-0.418	3.44E-06
**Sphingolipids - Sphingomyelin**				
SM (OH) C14:1	0.054	0.047	-0.698	5.31E-06
SM (OH) C16:1	0.074	0.068	-0.841	4.32E-08
SM (OH) C22:2	0.055	0.048	-0.828	4.40E-06
SM C16:0	0.051	0.045	-0.992	9.65E-06
**Monosaccharides**				
Sum of Hexoses (including Glucose)	0.065	0.058	1.368	4.05E-07

HOMA-IR and plasma metabolites are log transformed before analysis.

Linear regression model were adjusted for age and sex.

Bonferroni correction is applied to the multiple comparison for 141 metabolites (p<3.55E-04).

Supplementary Table S2. Linear regression between HOMA-IR and plasma metabolites in KoGES

Metabolites	***R^2***	**Adjusted *R^2***	**Beta**	**p-value**
**Acylcarnitines**				
Carnitine (C0)	0.003	0.002	0.093	5.93E-02
Decenoylcarnitine (C10:1)	-	-	-	-
Tetradecenoylcarnitine (C14:1)	0.002	0.000	-0.003	9.23E-01
Hydroxytetradecenoylcarnitine (C14:1-OH)	-	-	-	-
Tetradecadienylcarnitine (C14:2)	0.004	0.002	-0.044	4.82E-02
Hydroxytetradecadienylcarnitine (C14:2-OH)	-	-	-	-
Octadecenoylcarnitine (C18:1)	0.005	0.003	0.077	7.75E-03
Propionylcarnitine (C3)	0.023	0.022	0.223	2.50E-13
Pimelylcarnitine (C7-DC)	0.004	0.002	-0.058	2.53E-02
Nonaylcarnitine (C9)	-	-	-	-
**Amino-acids**				
Alanine	0.098	0.097	0.679	1.07E-56
Arginine	0.026	0.024	0.276	1.33E-14
Asparagine	0.006	0.005	-0.163	1.05E-03
Aspartate	0.003	0.002	0.052	8.82E-02
Glutamate	0.034	0.032	0.250	4.05E-19
Glycine	0.037	0.035	-0.415	6.08E-21
Isoleucine	0.054	0.052	0.536	1.89E-30
Leucine	0.037	0.035	0.494	7.40E-21
Lysine	0.002	0.001	0.047	3.27E-01
Phenylalanine	0.026	0.024	0.472	1.22E-14
Proline	0.044	0.042	0.391	6.52E-25
Tryptophan	0.002	0.001	0.053	2.88E-01
Tyrosine	0.055	0.053	0.578	3.61E-31
Valine	0.096	0.094	0.858	4.48E-55
**Biogenic Amines**				
Asymmetric dimethylarginine (ADMA)	0.005	0.004	0.078	4.46E-03
Creatinine	0.002	0.000	-0.066	1.64E-01
Kynurenine	0.007	0.005	0.128	5.85E-04
alpha-Aminoadipic acid	-	-	-	-
**Glycerophospholipids - Phosphatidylcholine (PC)**				
PC aa C32:1	0.025	0.023	0.154	3.11E-14
PC aa C36:1	0.021	0.019	0.229	5.65E-12
PC aa C36:3	0.010	0.008	0.165	1.40E-05
PC aa C38:0	0.006	0.005	-0.116	1.42E-03
PC aa C38:3	0.016	0.014	0.228	4.37E-09
PC aa C40:1	0.013	0.011	-0.194	2.71E-07

PC aa C40:2	0.012	0.011	-0.166	4.67E-07
PC aa C40:4	0.004	0.002	-0.068	3.75E-02
PC aa C40:5	0.017	0.015	0.161	1.39E-09
PC aa C42:0	0.030	0.028	-0.289	6.68E-17
PC aa C42:1	0.028	0.026	-0.295	1.14E-15
PC aa C42:4	0.024	0.022	-0.300	1.78E-13
**Glycerophospholipids - Phosphatidylcholine (PC)**				
PC ae C30:0	0.004	0.002	-0.082	3.53E-02
PC ae C32:1	0.004	0.002	-0.084	3.64E-02
PC ae C34:0	0.003	0.001	0.051	1.24E-01
PC ae C34:1	0.006	0.004	-0.127	2.98E-03
PC ae C34:3	0.045	0.044	-0.355	8.61E-26
PC ae C36:0	0.002	0.000	-0.003	9.16E-01
PC ae C36:2	0.011	0.009	-0.175	2.92E-06
PC ae C38:2	0.002	0.000	-0.015	6.44E-01
PC ae C40:1	0.022	0.020	-0.272	2.08E-12
PC ae C40:4	0.013	0.012	-0.231	9.23E-08
PC ae C40:5	0.003	0.002	0.074	5.85E-02
PC ae C40:6	0.008	0.006	-0.141	2.06E-04
PC ae C42:1	0.067	0.066	-0.507	2.05E-38
PC ae C42:2	0.014	0.012	-0.227	6.27E-08
PC ae C42:3	0.024	0.022	-0.328	1.37E-13
PC ae C42:4	0.039	0.038	-0.373	2.95E-22
PC ae C42:5	0.012	0.011	-0.242	3.42E-07
PC ae C44:5	0.017	0.015	-0.213	1.33E-09
PC ae C44:6	0.039	0.038	-0.345	2.09E-22
**Glycerophospholipids - lysoPC**				
lysoPC a C17:0	0.023	0.022	-0.251	2.60E-13
lysoPC a C18:1	0.021	0.019	-0.265	5.04E-12
lysoPC a C18:2	0.037	0.036	-0.325	4.16E-21
**Sphingolipids - Sphingomyelin**				
SM (OH) C14:1	0.016	0.015	-0.240	2.24E-09
SM (OH) C16:1	0.022	0.020	-0.270	1.41E-12
SM (OH) C22:2	0.039	0.037	-0.389	4.44E-22
SM C16:0	0.045	0.043	-0.549	2.24E-25
**Monosaccharides**				
Sum of Hexoses (including Glucose)	0.203	0.202	1.081	2.18E-123

HOMA-IR and plasma metabolites are log transformed before analysis.

Linear regression model were adjusted for age, sex and region.

Supplementary Table S3. Clinical characteristics of obesity intervention program subjects (n=91)

	Baseline		Follow-up		change over 10 weeks		p-value
Age (years)	14.0	± 0.83					
Boys [n (%)]	46	(50.5)					
Height (cm)	164.8	± 7.44	164.9	± 7.48	0.07	± 0.24	**0.010**
Weight (kg)	92.7	± 13.4	92.7	± 14.8	0.01	± 3.15	0.979
BMI (kg/m^2)	34.0	± 2.95	33.9	± 3.40	-0.05	± 1.13	0.676
BMI z-score	2.79	± 0.37	2.77	± 0.40	-0.02	± 0.14	0.279
Fat mass (kg)	41.5	± 8.31	41.8	± 9.20	0.26	± 3.38	0.469
Fat free mass (kg)	50.4	± 7.36	49.9	± 7.18	-0.48	± 2.68	0.094
Waist circumference (cm)	103.6	± 9.15	101.2	± 10.1	-2.41	± 5.09	**<0.001**
Hip circumference (cm)	112.7	± 6.19	111.8	± 6.76	-0.89	± 2.86	**0.004**
WHR (waist-to-hip ratio)	0.92	± 0.05	0.90	± 0.06	-0.01	± 0.04	**0.001**
Systolic blood pressure (mmHg)	125.7	± 14.7	120.9	± 12.1	-4.73	± 11.4	**<0.001**
Diastolic blood pressure (mmHg)	78.8	± 9.20	76.6	± 9.09	-2.20	± 9.38	**0.028**
Glucose (mg/dL)	95.4	± 18.6	99.6	± 24.5	4.24	± 13.4	**0.004**
AST (IU/L)	29.3	± 17.5	26.3	± 20.4	-3.08	± 14.3	**0.045**
ALT (IU/L)	40.8	± 38.4	35.3	± 37.7	-5.52	± 16.9	**0.003**
Total cholesterol (mg/dL)	178.6	± 26.5	175.8	± 29.7	-2.76	± 21.5	0.229
Triglyceride (mg/dL)	132.5	± 71.2	118.1	± 56.7	-14.4	± 57.0	**0.019**
HDL-cholesterol (mg/dL)	44.5	± 7.10	46.3	± 8.35	1.75	± 6.99	**0.020**
ADMA	0.57	± 0.16	0.43	± 0.14	-0.14	± 0.18	**<0.001**

Values are expressed as the mean±SD or n (%).

BMI, body mass index; HDL, high density lipoprotein; HOMA-IR, homeostasis model assessment insulin resistance; ADMA, asymmetric dimethylarginine.

Supplementary Fig. S1. Molecular pathway networks interacting with metabolites.

A. Overall networks

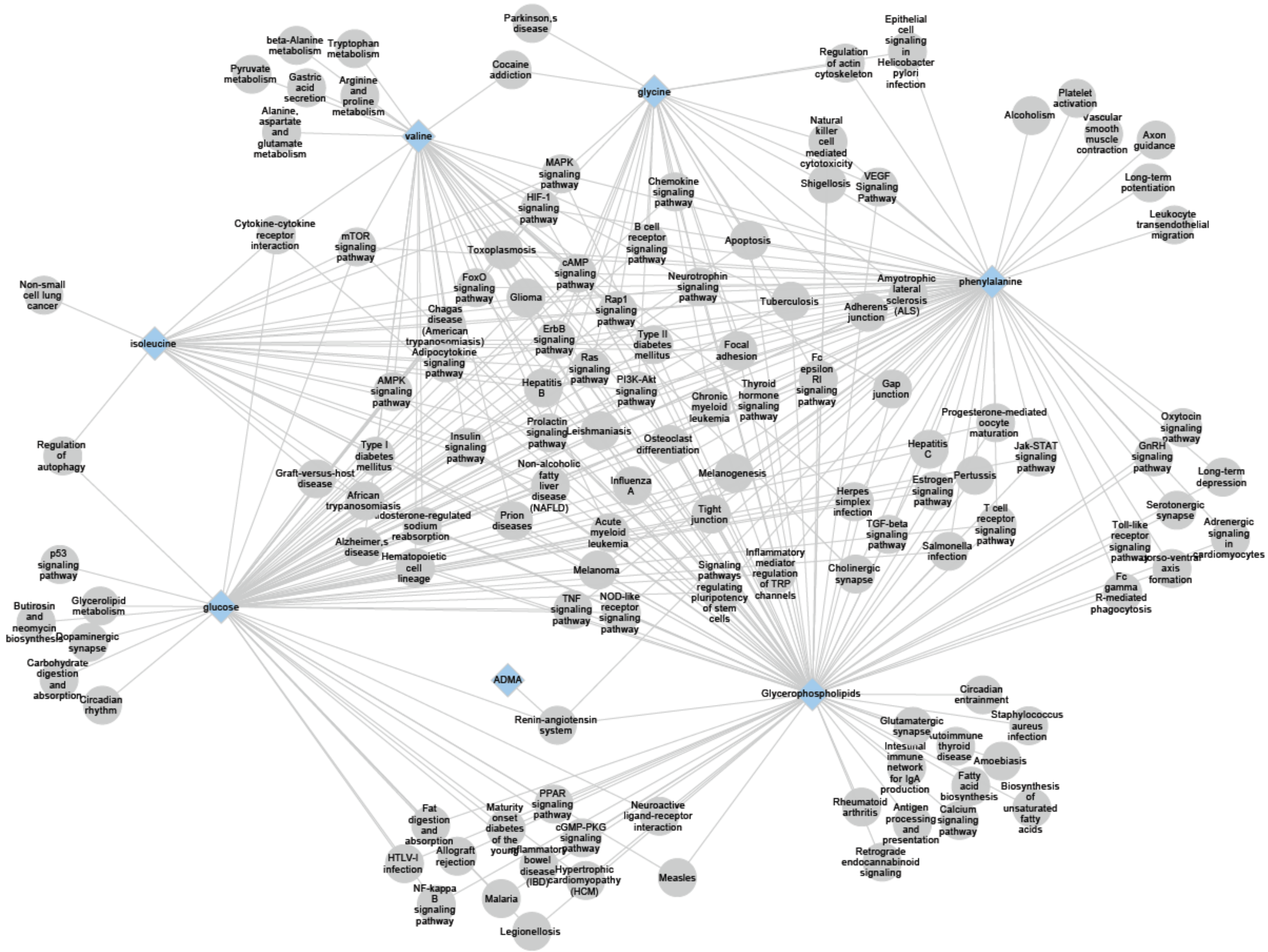

B. Simplified interaction network between insulin resistance and metabolites

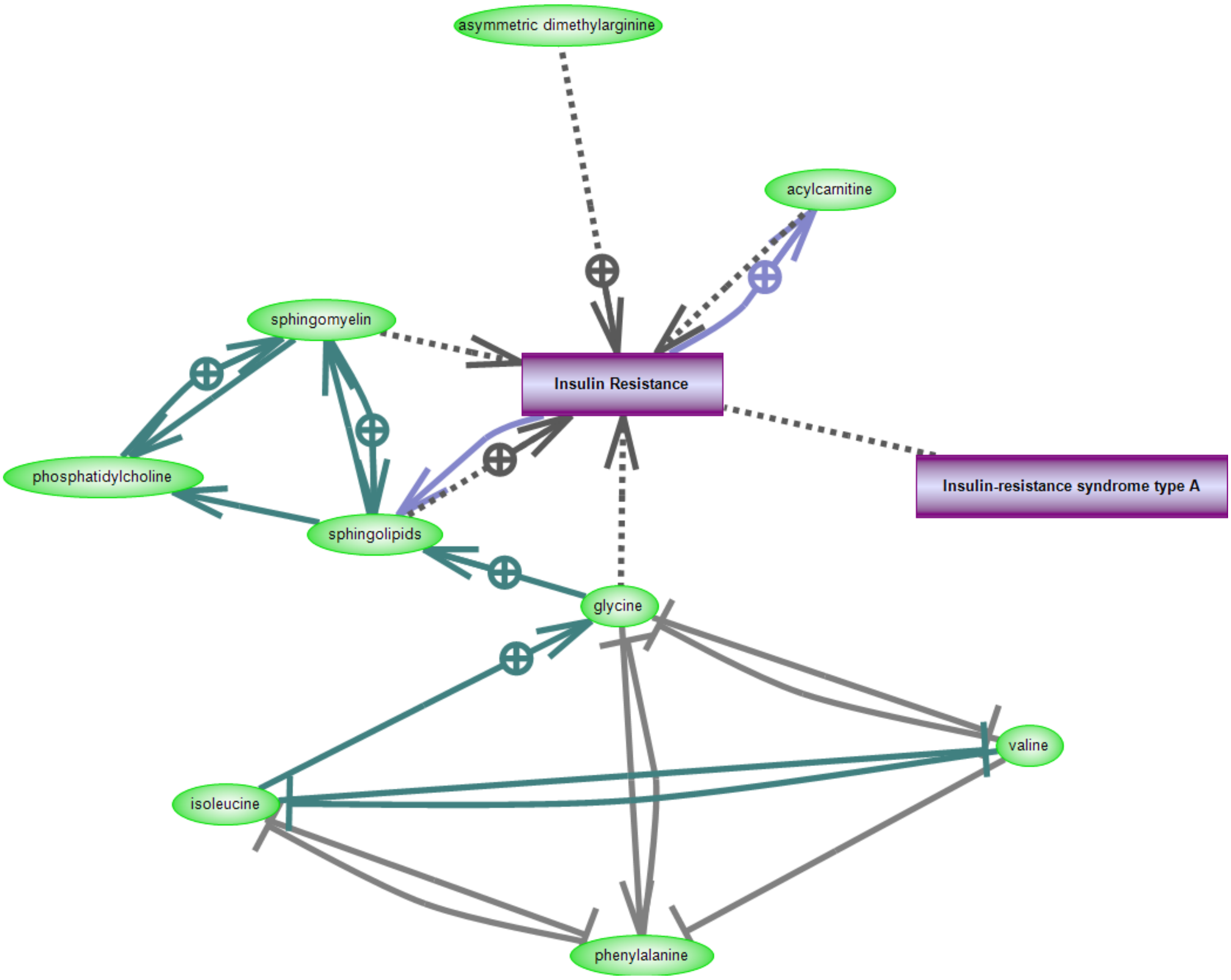

Supplement: Supplementary file 1 — Supplementary Information [file 41598_2018_20549_MOESM1_ESM.pdf]
